# Fruit Phenolic and Triterpenic Composition of Progenies of *Olea europaea* subsp. *cuspidata*, an Interesting Phytochemical Source to Be Included in Olive Breeding Programs

**DOI:** 10.3390/plants11141791

**Published:** 2022-07-06

**Authors:** Irene Serrano-García, Lucía Olmo-García, Daniel Polo-Megías, Alicia Serrano, Lorenzo León, Raúl de la Rosa, Ana María Gómez-Caravaca, Alegría Carrasco-Pancorbo

**Affiliations:** 1 Department of Analytical Chemistry, Faculty of Sciences, University of Granada, Ave. Fuentenueva s/n, 18071 Granada, Spain; iserrano@ugr.es (I.S.-G.); danielpm@ugr.es (D.P.-M.); anagomez@ugr.es (A.M.G.-C.); alegriac@ugr.es (A.C.-P.); 2 IFAPA Centro Alameda del Obispo, Ave. Menéndez Pidal s/n, 14004 Córdoba, Spain; alicia.serrano.gomez@juntadeandalucia.es (A.S.); lorenzo.leon@juntadeandalucia.es (L.L.); raul.rosa@juntadeandalucia.es (R.d.l.R.)

**Keywords:** breeding programs, *cuspidata*, olive drupe, metabolic profile, LC-MS

## Abstract

*Olea europaea* subsp. *cuspidata* has a relatively low commercial value due to the low size and pulp to stone ratio of its drupes compared to commercial olive cultivars. Nevertheless, this subspecies could represent a valid source of useful traits for olive breeding. In the current work, the drupe metabolic composition (secoiridoids, flavonoids, simple phenols, triterpenic acids, etc.) of a progeny of 27 *cuspidata* genotypes coming from free pollination and their female parent was evaluated by applying a powerful LC-MS method. A total of 62 compounds were detected within the profiles; 60 of them were annotated and 27 quantified. From a quantitative point of view, the genotypes from the progeny of *cuspidata* showed quite different metabolic profiles to olive common cultivars (“Arbequina”, “Frantoio”, “Koroneiki” and “Picual”) used as controls. *Cuspidata* drupes were richer in terms of several bioactive compounds such as rutin, hydroxytyrosol glucoside, a few interesting secoiridoids and the compounds of *m*/*z* 421 and 363. The relationships among several secondary metabolites determined in the progeny inferred from the results of both PCA and cross-correlation analysis were explained according to metabolic biosynthesis pathways in olive drupes. These outcomes underlined the potential of *cuspidata* genetic resources as a source of potentially interesting variability in olive breeding programs.

## 1. Introduction

The genus *Olea* belongs to the family *Oleaceae* and is divided into three different subgenera: *Olea*, *Tetrapilus* and *Paniculatae* [1]. Six subspecies have been defined for *Olea europaea* L., which is popularly known as “The Olive Complex”. The subsp. *europaea* (diploid), which can be found throughout the whole Mediterranean basin, is represented by two botanical varieties: cultivated olive (*Olea europaea* subsp. *europaea* var. *europaea*) and wild olive (*Olea europaea* subsp. *europaea* var. *sylvestris*). Additionally, five more non-cultivated sbspp. have been described: *laperrinei* (diploid), *cuspidata* (diploid), *guanchica* (diploid), *maroccana* (polyploid 6n) and *cerasiformis* (polyploid 4n) [2,3,4]. The geographical origin and domestication of olive tree remain unclear. It is usually accepted that olive tree domestication began in the Northern Levant approximately six thousand years ago [5]. Different paleobotanic and genetic investigations have hypothesized that the current cultivars arose from one or multiple random hybridizations between wild and domesticated Mediterranean genotypes. Both wild and cultivated olive trees have coexisted in human civilizations [4,6].

Nowadays, the cultivated olive tree is considered the most emblematic tree of the Mediterranean basin and is of undeniable economic importance. Spain tops the list of major olive-producing countries with an annual production of almost 10 million tonnes, followed by Italy and Greece with productions of approximately 1.9 and 1.1 million [7]. Meanwhile, *Olea europaea* subsp. *cuspidata*, also called *Olea ferruginea Royle* (the wild non-Mediterranean olive), has a wide continental distribution from Austral Africa to China. Its presence has also been reported in Australia, north of New Zealand and Hawaii [2]. It has been hypothesized that the subsp. *cuspidata* ancestors contributed to the origin of cultivated olive. The African olive (subsp. *cuspidata*) is not of a great economic importance due to its little drupe size (diameter generally < 8 mm). Nevertheless, it could represent a valid source of useful traits for cultivated olive, such as adaptability to semi-arid to *meso*-humid climate conditions and resistance to abiotic or biotic stresses [8]. Its wood is used to make furniture or as vegetable hedge, while leaves and other plant organs are locally used for the treatment of various diseases [9,10,11]. Both subspecies (*europaea* and *cuspidata* subsp.) are sexually compatible either in nature or in experimental crosses, which could be particularly interesting for the introgression of some agronomic traits, phytochemical features and/or resistance to biotic and abiotic stresses in breeding programs.

Olive breeding initiatives have been developed in several countries around the world (Argentina, Australia, Croatia, France, Greece, Iran, Israel, Italy, Jordan, Lebanon, Montenegro, Morocco, Portugal, Spain, Tunisia, Turkey, Uruguay, and USA) [12,13]. Breeding programs are focused on improving agronomic traits such as early bearing, productivity, oil content and composition [14,15,16]. Tolerance to abiotic and biotic stresses such as *Verticillium dahliae* or *Xylella fastidiosa* are also important objectives for olive breeding programs [17]. Pérez and collaborators proposed a high-throughput methodology to include the phenolic composition as a selection criterion in olive breeding programs [18]. The high correlation between fruit and the content of oil phenolic components and the high genotypic variance described for these compounds suggest the usefulness of the analysis of fruit phenolic compounds in olive breeding programs to select olive genotypes of potential interest in terms of oil phenolic composition.

The metabolic profile of wild olives has not been explored and is practically unknown; on the contrary, there is a lot of research focused on the study of the minor fraction of common cultivated olives. The minor fraction of olive fruit represents approximately 1 to 3% of the total olive composition and contains, among others, phenolic compounds, pentacyclic triterpenes, tocopherols and phytosterols [19]. The phenolic fraction is very complex and its profile is conditioned by many factors (cultivar, ripening stage, season, etc.); it comprises secoiridoids, simple phenols, phenolic acids, flavonoids and lignans. The potent antioxidant activity, beneficial health effects and influence on sensory characteristics of olive oil are some of the properties that have been ascribed to these compounds [20,21]. Pentacyclic triterpenes are mainly found in the stem bark and in the surface cuticular waxes of olive leaves and fruits. The most studied ones are the maslinic, oleanolic and ursolic acids and the alcohols erythrodiol and uvaol. Numerous health-promoting properties have been attributed to them [22,23,24]. Tocopherols and phytosterols are mainly present in olive oil and their intake is related, among other factors, to the protective capacity against oxidative stress and the regulation of cholesterol, respectively [25,26]. The assessment of the mentioned minor compounds in olive cultivars (in particular, phenolic compounds and triterpenes) has been traditionally addressed by studying each family of compounds separately (i.e., by using targeted approaches). Table 1 includes some of the most comprehensive reports describing olive fruit’s minor components. Information about the cultivar, analytical platform(s) used, determined compounds, etc., has been gathered within the table; when the studies considered more than one olive-derived matrix, it has been pointed out.

To date, there are only few studies dealing with the characterization of different olive oils obtained from wild olives from various origins (Pakistan, Tunisia, Algerian or Portugal) [34,35,36,37,38,39]. Dabbou and co-authors, for instance, observed that oleasters could be potentially interesting, since they produced oils with good quality characteristics in terms of minor compounds (phenols and volatiles) compared to the “Chemlali Sfax” cultivar [38]. Similarly, Bouarroudj and colleagues highlighted the high potential of Algerian oleaster oils as phytochemical and genetic resources to improve the quality of olive oil [37]. Another thorough study has suggested that the use of wild germplasm in olive breeding programs will not have a negative impact on olive oil composition in terms of fatty acids, tocopherol content and tocopherol and phytosterol profiles, given that the selection of these compounds is conducted starting from early generations [15]. Unfortunately, the potential of *cuspidata* olive drupes regarding their phytochemical composition has not yet been deciphered and their differences with cultivated olives have been scarcely studied.

Therefore, the objective of the present work was: (i) to perform an in-depth characterization of the metabolic profile of *cuspidata* samples; (ii) to compare their compositional profiles regarding phenolic and triterpenic substances (qualitatively and quantitatively) with that of four olive common cultivars (“Arbequina”, “Frantoio”, “Koroneiki” and “Picual”); and (iii) to evaluate whether the subsp. *cuspidata* could represent a valid source of useful traits for cultivated olive, proving eventually the potential of this subspecies to be included in breeding programs.

## 2. Results and Discussion

### 2.1. Characterization of the Metabolic Profile of Progenies from Olea europaea *subsp.* cuspidata by LC-MS

As stated in the Materials and Methods (see Section 3), liquid chromatography (LC) coupled with high-resolution mass spectrometry (HRMS) was used to perform a qualitative profiling of the extracts of the subsp. *cuspidata* fruit samples. A total of 62 compounds were detected within the profiles; a combination of accurate mass and isotopic distribution was used to calculate the theoretical elemental formula of the detected metabolites. The identity of some compounds was verified by using the commercial or isolated pure standards available in-house; for some other metabolites, however, we just provided a tentative identification based on a combination of experimental data (HRMS data and *in-source* fragmentation patterns), the expertise of our research group and the information previously described in the literature regarding olive fruit characterization [28,30,31,33]. Table 2 shows the qualitative exploration of progenies from *Olea europaea* subsp. *cuspidata*. Each row of the table includes the identity assigned to each analyte, to which chemical class it might belong, its molecular formula, retention time, experimental and theoretical *m*/*z* signals, error (ppm) and mSigma value, as well as the *in-source* fragments detected in MS.

Secoiridoids (40) made up the most numerous group of compounds, followed by flavonoids (10), pentacyclic triterpenes (5), simple phenols or related analytes (3) and organic acids (2). It should be noted that a large part of the identified compounds corresponded to glycosylated derivatives and isomers, especially in the case of secoiridoids. As far as secoiridoids are concerned, 22 analytes were structurally related to hydroxytyrosol (oleuropein derivatives), 3 to tyrosol (ligstroside derivatives) and 12 resulted to be oleoside-type and elenolic acid derivatives. In many of the genotypes evaluated, the compounds annotated as oleuropein, verbascoside, elenolic acid glucoside (isomer C), demethyl oleuropein, lucidumoside C, ligstroside and oleoside/secologanoside (isomer C) were the peaks with the highest relative intensity in the profiles. Similarly, several oleuropein-, ligstroside-, and elenolic acid-derived compounds, such as oleuropein aglycone isomers, demethyl ligstroside and acyclodihydroelenolic acid hexoside (isomer B), were found to be relevant in the chromatographic profile of the *cuspidata* samples. The presence of oleuropein and ligstroside aglycones in the drupes is the consequence of the overexpression of the *β*-glucosidase enzyme, which is involved in the ripening mechanism [28]. In this case, two isomers of oleuropein aglycon and some of its derivatives (dehydro oleuropein aglycone A and B, hydroxy decarboxymethyl oleuropein aglycone, and 10-hydroxy oleuropein aglycon A and B) were detected in *cuspidata* samples, while ligstroside aglycones were not detected.

The second most numerous group of compounds was flavonoids. In this category, we found the following substances: rutins A and B, luteolin 7-O-glucoside, luteolin rutinoside, luteolin glucoside isomers A, B and C, apigenin 7-O-glucoside, luteolin and apigenin. Luteolin 7-O-glucoside and luteolin glucoside isomer B (*m*/*z* 447.0937) and, in particular, rutin (*m*/*z* 609.1463) were the most abundant ones.

Within the category of pentacyclic triterpenes, five compounds were identified: maslinic acid (*m*/*z* 471.3479), betulinic acid (*m*/*z* 455.3529), oleanolic acid (*m*/*z* 455.3528), an isomer with *m*/*z* 455.3531 and a monohydroxylated derivative of maslinic acid. These compounds have been previously reported by other authors in olive fruit tissues of subspecies *europaea* [23,33].

Substances belonging to the chemical classes of simple phenols and organic acids were also found in the LC-MS profiles of *cuspidata* genotypes. Regarding simple phenols (or similar compounds), three compounds were identified: hydroxytyrosol glucoside (*m*/*z* 315.1081), oxydized hydroxytyrosol (*m*/*z* 151.0395) and phenylethyl primeveroside (*m*/*z* 415.1606). Organic acids were the most polar analytes of all those detected in the profiles, eluting at the beginning of the chromatogram. Within this category, quinic and citric acids were found in the samples. Only the first one (*m*/*z* 191.0550) was remarkable due to its intensity in the profile.

Three other substances, which were found in the profiles with high relative intensities, could not be identified with confidence. The peak with *m*/*z* 537.1605 (C_25_H_30_O_13_) was tentatively assigned to fraxamoside, considering that its presence has been recently described in Greek olives by Kritikou and co-authors [40]. The MS/MS analysis described in their work agreed with some of our *in-source* fragments (*m*/*z* 323.0811 and 221.0273), which suggests that it could be the same compound they described. The second unknown peak was the one with *m*/*z* 363.1440, which could be a compound related to ligstroside aglycone (the predicted molecular formula was C_19_H_24_O_7_), and the third one was the peak with *m*/*z* 421.1494 and molecular formula C_21_H_26_O_9_. Our hypothesis regarding the latter one is that it could be a secoiridoid derivative (oleuropein aglycone + C_2_H_4_O or ligstroside aglycone acetate). Some experiments are already in progress to be able to assign an identity to them in the near future.

### 2.2. Application of LC-MS for the Quantitative Evaluation of Samples under Study

**Figure 1:**
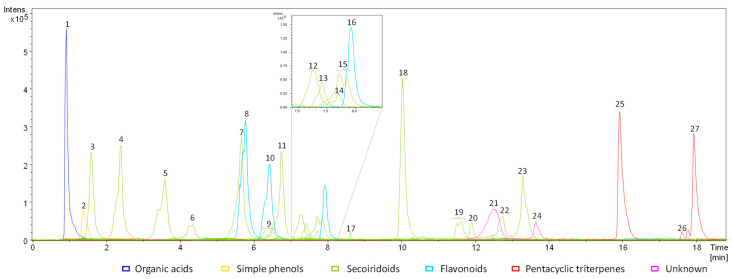
Example of the profile obtained for the extract of one *cuspidata* sample (316-6-G14) including the chromatograms of the extracted ions of the quantified metabolites. Colors have been used to indicate belonging to the different chemical categories of each numbered peak: organic acids (dark blue), simple phenols (yellow), secoiridoids (green), flavonoids (light blue), pentacyclic triterpenes (red) and unknown compounds (pink).

From the identified compounds, a total of 27 metabolites were quantitatively assessed in the samples under study by using LC coupled to low-resolution (LR) MS (Figure 1). The choice of the compounds to be quantified was mainly based on: (1) the compounds having a higher prevalence (in terms of area and intensity; i.e., they are more abundant) in the chromatographic profiles, and (2) having an appropriate pure standard to perform a proper quantification. We decided to quantify three flavonoids (luteolin glucoside (isomer B), luteolin 7-O-glucoside and rutin (isomer B)), one organic acid (quinic acid), three pentacyclic triterpenes (betulinic, oleanolic and maslinic acids), sixteen secoiridoids (caffeoyl 6-secologanoside, dihydro oleuropein, dehydro nuzhenide, *β*-hydroxy verbascoside, neonuzhenida, methoxy oleuropein (isomer A), oleuropein aglycone isomers A and B, demethyl ligstroside, acyclodihydroelenolic acid hexoside (B), oleoside/secologanoside (isomer C), ligstroside, lucidumoside C (isomer A), demethyl oleuropein, elenolic acid glucoside (isomer C), verbascoside and oleuropein), one simple phenol (hydroxytyrosol glucoside) and two unknown compounds, with *m*/*z* of 363 and 421, respectively. Most of the secoiridoids and the two unknown compounds were quantified in terms of oleuropein. *β*-hydroxy verbascoside, verbascoside, caffeoyl 6-secologanoside and demethyl ligstroside were quantified by using the calibration curve obtained with the pure standard of verbascoside. This seemed appropriate because a relatively low area was found for the latter compounds in the samples under study. Hydroxytyrosol glucoside was quantified with the hydroxytyrosol standard, and luteolin glucoside in terms of its isomer luteolin 7-O-glucoside.

### 2.3. Comparison between Cuspidata and Cultivars Fruits: Evaluating the Potential of Cuspidata Phytochemical Source to Be Included in Olive Breeding Programs

#### 2.3.1. Fruit Weight, Oil Content and Total Compounds of Wild and Cultivated Olives

**Figure 2:**
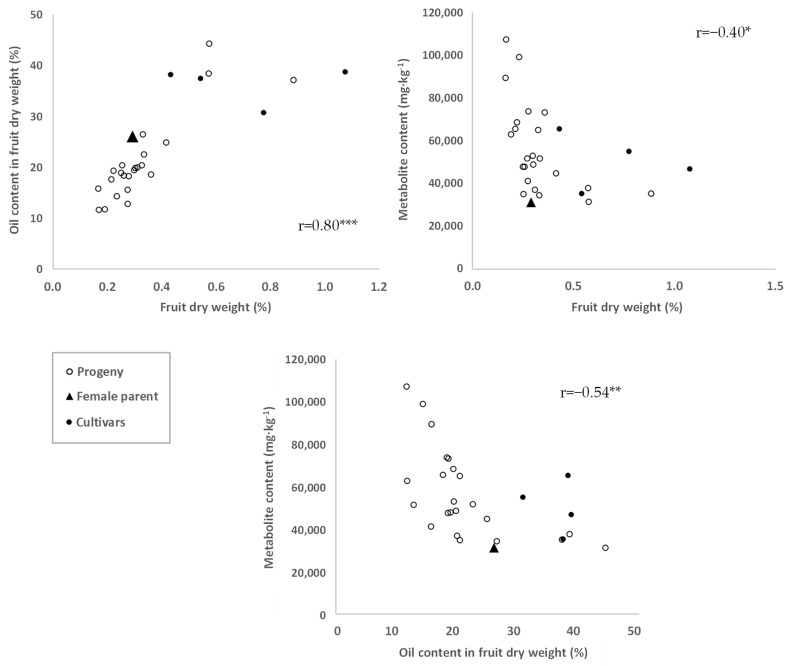
Evaluation of the relationships between fruit weight (g), oil content (%) and total metabolite content (mg·kg^−1^). *, **, ***: significant at *p* < 0.05, 0.01, 0.001, respectively.

As expected, a highly significant correlation was found between fruit weight and oil content (r = 0.80, *p* < 0.001), with most *cuspidata* genotypes and their female parents, in the lower range of values for these two traits, respect to the four cultivars analyzed (Figure 2, upper left). The oil yield ranged from 10 to 45% approx. (fruit dry weight (%)) in *cuspidata* fruit, although most genotypes exhibited values between 10 and 25%. Similar contents were reported by Joshi and Gulfraz et al., ranging from about 20 to 28% for *Olea ferruginea Royle* in the north-west of India and from 33 to 39% in Pakistan, respectively [41,42]. Another study described lower values of oil yield by mill extraction for *Olea ferruginea Royle* from Pakistan, within the range from 11.1 to 12.5% [34]. A few *cuspidata* genotypes showed values for these two traits close to the ones obtained for the cultivars, which indicates that potentially interesting values for these attributes can be recovered in a single generation.

The relationship between fruit weight or oil content and total metabolite content (right and lower parts of Figure 2) was not so clear, even though a significant negative correlation was observed in both cases. Similar results were obtained also for either individual components or different chemical categories (data not shown). Higher contents of some others minor compounds such as tocopherols, associated with concomitant lower values for fruit size and oil content has been also reported in non-cultivated olive plant materials [15]. Additionally, this negative relationship is always found with lower values for phenolic and other minor components as fruit size and oil content increase during fruit ripening [30,43].

#### 2.3.2. Quantitative Evaluation of the Selected Individual Compounds and Principal Component Analysis to Explore the Natural Clustering of the Samples

Table 3 presents a summary of the quantitative data. The quantitative data for each and every compound quantified in the progeny, the female parent and the cultivars have been included in Table S1 (Supplementary Material). Two independent replicates of each *cuspidata* genotype and cultivar samples (n = 28 × 2 (*cuspidata*) and n = 4 × 2 (cultivars), respectively), injected twice, were used to obtain the final quantitative values.

As observed in Table 3, most of the 27 compounds selected to be quantified were determined in all the genotypes of the *cuspidata* progeny, with the exception of methoxy oleuropein, demethyl ligstroside, ligstroside and demethyl oleuropein, which were quantified in 27 samples; luteolin 7-O-glucoside and *β*-hydroxy verbascoside, which were determined in 26 samples; and verbascoside, which was only quantified in 18 wild olive fruit extracts. Metabolites that were not found in all samples of *O. europaea* subsp. *europaea* were *β*-hydroxy verbascoside, verbascoside and the unknown compound with *m*/*z* 363, quantified in three of the four cultivars; methoxy oleuropein (A) and demethyl oleuropein, determined in two cultivars (“Arbequina” and “Frantoio”); and neonuzhenide and demethyl ligstroside, which were only quantified in “Frantoio”.

The main differences between the pulp of *cuspidata* and *europaea* samples appeared to be associated with flavonoids, particularly rutin. It was the most abundant flavonoid in both types of samples, but its concentration in *cuspidata* was five times higher than in the cultivars. The organic acids and pentacyclic triterpenes exhibited similar concentrations in the two types of samples and simple phenols were higher in *cuspidata* pulp, but not by much. Although, in the secoiridoid family, verbascoside and oleuropein were the predominant metabolites for both progeny and conventional olives, some differences were observed.

Substances such as demethyl ligstroside, oleoside/secologanoside (C), ligstroside, lucidumoside C (A), demethyl oleuropein and elenolic acid glucoside (C) were consistently more abundant in wild olives, whereas, for instance, dihydro oleuropein, oleuropein aglycone (isomers A and B) and acyclodihydroelenolic acid hexoside (B) were, on average, more abundant in cultivars.

**Figure 3:**
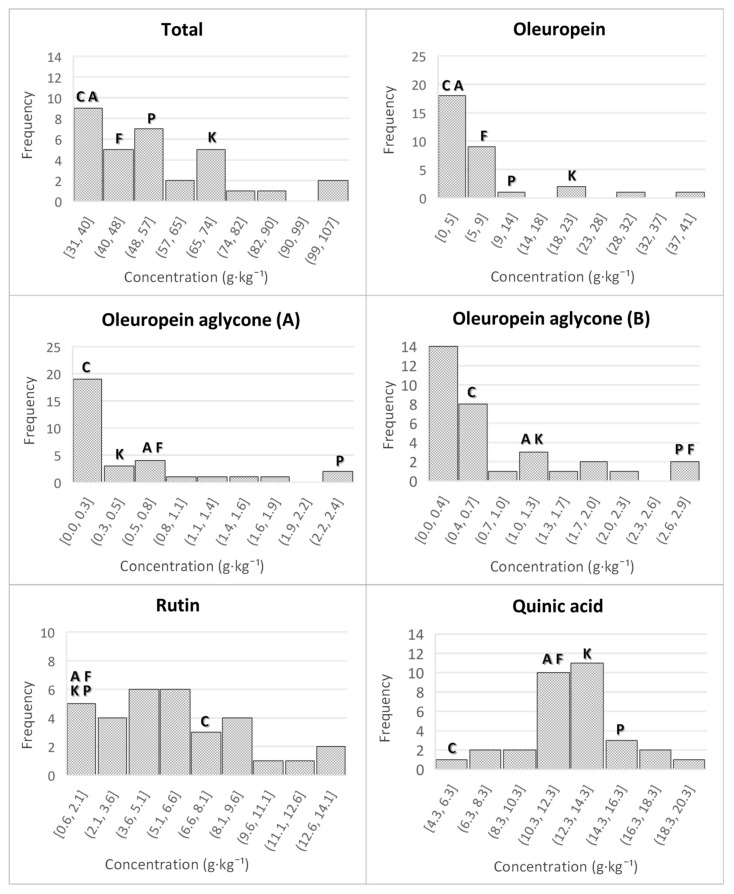
Histograms for total metabolite content and five specific compounds analyzed in the progeny (g·kg^−1^). The ranges in which the female parent and cultivars fall are indicated with letters as follows: female parent of the open pollination progeny—C; “Arbequina” cultivar—A; “Frantoio” cultivar—F; “Koroneiki” cultivar—K; “Picual” cultivar—P.

Figure 3 shows the quantitative distribution of some compounds in the samples of progeny, the female parent of open pollination progeny and the cultivars. In all cases, the x-axis shows the concentration in g·kg^−1^ and the y-axis the frequency (the number of samples that exhibited concentrations in a given range); letters (to facilitate interpretation) indicate in which group the female parent or the different cultivars fell. The range of variability for the *cuspidata* progeny markedly expands the value of their corresponding female parents for total metabolite contents, achieving, therefore, a huge improvement in one single generation. Cultivars showed intermediate ranges of total metabolite concentration, while the highest values were found for some *cuspidata* samples.

When the oleuropein histogram was studied in detail, it was noted that most of the wild progeny clustered together with their female parents in the lowest range of concentration, although some exceptional genotypes showing high oleuropein contents were also obtained among the *cuspidata* progeny. Oleuropein aglycone (isomers A and B) prevailed in cultivars, especially in “Picual” and “Frantoio”; although there were some *cuspidata* samples that were richer than the cultivars, the most common situation was that the greatest number of genotypes fell into the lower concentration ranges (together with the female parent). Rutin showed a histogram quite different from those just discussed. “Arbequina”, “Frantoio”, “Koroneiki” and “Picual” exhibited the lowest concentrations; the female parent of the progeny, however, showed concentrations at least three times higher than those of the cultivars. Eleven genotypes had rutin levels equal to or higher than those of the female parent (reaching values of up to 14.1 g·kg^−1^) and all were substantially richer than the cultivars. The quinic acid content of the progeny appeared to be comparable to that of the cultivars ranging from 10.3 to 14.3 g·kg^−1^ for 18 of the genotypes studied.

**Figure 4:**
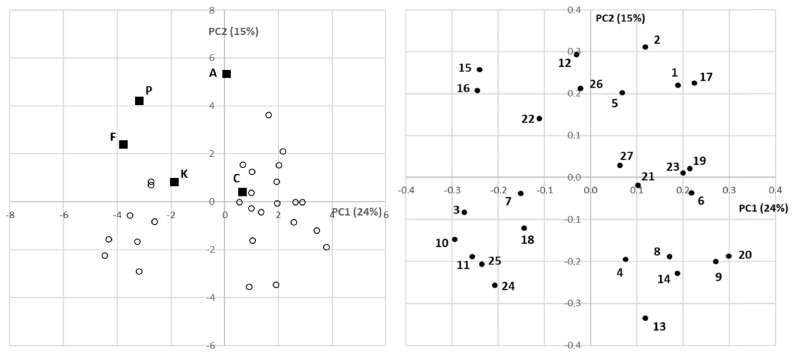
Score (**left**) and loading (**right**) plots from PCA. Meaning of letters in the score plot: female parent of the open pollination progeny, C; ”Arbequina” cultivar—A; “Frantoio” cultivar—F; “Koroneiki” cultivar—K; “Picual” cultivar—P. Meaning of numbers in the loading plot: 1—quinic acid; 2—maslinic acid; 3—oleuropein; 4—rutin (B); 5—oleanolic acid; 6—hydroxytyrosol glucoside; 7—verbascoside; 8—elenolic acid glucoside (C); 9—demethyl oleuropein; 10—ligstroside; 11—lucidumoside C (A); 12—acyclodihydroelenolic acid hexoside (B); 13—oleoside/secologanoside (C); 14—demethyl ligstroside; 15—oleuropein aglycone (B); 16—oleuropein aglycone (A); 17—luteolin 7-O-glucoside; 18—unknown 2 (*m*/*z* 421); 19—unknown 1 (*m*/*z* 363); 20—methoxy oleuropein (A); 21—neonuzhenide; 22—dihydro oleuropein; 23—luteolin glucoside (B); 24—*β*-hydroxy verbascoside; 25—dehydro nuzhenide; 26—caffeoyl 6-secologanoside; 27—betulinic acid.

Some of the details just mentioned were also revealed in the principal component analysis (PCA), which was used to perform a preliminary exploratory analysis of the variability between and within the groups of samples evaluated (Figure 4). The PCA score plots obtained using the entire LC-MS quantitative data set are displayed in a two-dimensional plot using the first two principal components (left of Figure 4), which covered 24.0% and 15.0% of the total variance, respectively. The graph shows a quite clear separation among the cultivars and the genotypes from the progeny, although the *cuspidata* samples were spread over several areas of the plot, which would mean that a relatively wide range of variability was observed over the entire progeny. The right part of Figure 4 shows the loading plots of the PCA model. The meaning of the numbers assigned to each compound is shown in the figure caption; these were assigned considering the relative abundance (in decreasing order) in the progeny samples.

PC1 correlated positively, mainly, with hydroxytyrosol glucoside, unknown 1 (*m*/*z* 363), neonuzhenide, luteolin glucoside (B) and betulinic acid, and negatively with oleuropein and verbascoside. PC2 was positively related to oleanolic acid, acyclodihydroelenolic acid hexoside (B) and caffeoyl 6-secologanoside, among other compounds.

In view of the loading plot, it can be stated that five secoiridoids are able to define a fairly typical pattern for samples of *O. europaea* subsp. *europaea*, with these substances being the following: dihydro oleuropein, acyclodihydroelenolic acid hexoside (B), caffeoyl 6-secologanoside and isomers A and B of oleuropein aglycone.

#### 2.3.3. Quantitative Results Structured by Chemical Classes

**Figure 5:**
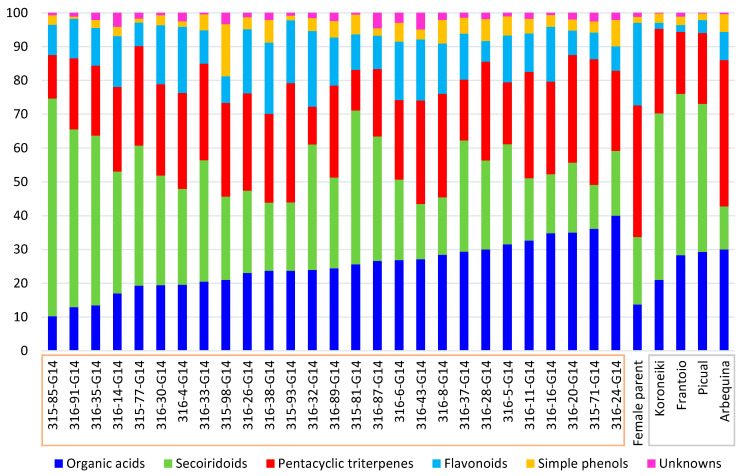
Description of the compositional pattern of each sample according to the percentage that each family of compounds represents with respect to the total concentration of metabolites (with all values normalized to the maximum metabolite concentration found for each sample). All the samples of the progeny are on the left side of the graphic (G-14) and the female parent in between the progeny and cultivars.

In this section, we intend to discuss the results considering the different families of metabolites that were determined, i.e., flavonoids, organic acids, triterpenes, secoiridoids, simple phenols and unknowns. For this purpose, Figure 5 shows a graph describing the compositional pattern of each sample according to the percentage that each family of compounds represents with respect to the total concentration of metabolites (with all values normalized to the maximum metabolite concentration found for each sample).

“Koroneiki”, “Frantoio” and “Picual” seem to have a percentage distribution of the different chemical classes quite comparable to each other. “Arbequina” was not found to have a very similar compositional distribution to the other cultivars, showing the highest percentage of simple phenols (5.3%), pentacyclic acids (43.3%) and flavonoids (8.4%).

The relative abundance of the different families of compounds in the female parent is not comparable with any of the cultivars. A great content of flavonoids (6.1–22.4%) was observed in the progeny, although none of the evaluated *cuspidata* genotypes exceeded the percentage of flavonoids found in the female parent (24.4%). Simple phenols and pentacyclic triterpenes ranged from 0.7 to 15.4% and 11.2 to 37.2%, respectively, in the wild olive extracts. Organic acids and secoiridoids showed the highest overall and maximum percentages in the genotypes of the progeny, ranging from 10.2 to 40.0% and 13.0 to 64.5%, respectively. It would be possible to establish a hypothetical correlation between these two families, since, in general, the lower the percentage of quinic acid in a sample, the higher the percentage of secoiridoids found.

#### 2.3.4. Preliminary Exploration of Metabolic Pathways: Cross-Correlation of the Secondary Metabolites Determined in the Progeny

The metabolic biosynthesis pathways in olive matrices are exceptionally complex. A great diversity in the structures and dynamic transformations of compounds are found during development, ripening, harvesting or olive oil extraction. Different pathways, including the shikimate, phenylpropanoid, mevalonate and flavonoid pathways, have been described as the basis for producing several precursors of phenolic compounds. Briefly, the shikimate pathway consists of the condensation of phosphoenolpyruvic acid and erythrose-4-phosphate to synthesize 3-dehydroquinic acid, which is transformed into shikimic acid. The final metabolite known as chorismic acid is synthesized in subsequent reactions and is a key branch point for the formation of L-Phenylalanine, which is the substrate of phenylpropanoid and flavonoid pathways [44]. Secoiridoids, the main iridoids found in Oleaceae, are biosynthesized by the mevalonate pathway from deoxylorganic acid. The connection of secoiridoids to the shikimate pathway is provided by two simple phenols (tyrosol and hydroxytyrosol) synthesized in the phenylpropanoid pathway [45,46,47]. For example, oleuropein is synthesized from hydroxytyrosol, which in turn is also related to ligstroside.

A cross-correlation for the metabolites determined in the progeny is shown in Table 4; it was carried out to evaluate whether a certain metabolic relationship could be established between some of the compounds under study in the present investigation. This table shows a positive significant correlation (*p* < 0.001) between luteolin glucoside and luteolin 7-O-glucoside, as well as oleuropein aglycones A and B, respectively. A balance in the synthesis of isomeric compounds could be the most plausible reason for these correlations. Likewise, a dynamic interconversion between some secoiridoids could be the cause of the significant positive correlation (*p* < 0.001) highlighted for some compounds in the cross-correlation table. The correlation noted in this table between dimethyl oleuropein and the unknown *m*/*z* 363 leads us to think that this compound could be a secoiridoid. Since its predicted molecular formula is C_19_H_24_O_7_, we hypothesize that it is a substance possibly related to ligstroside aglycone (perhaps with one less double bond).

In addition, quinic acid showed strong and inverse correlations (*p* < 0.001) with oleuropein and lucidumoside C (isomer A). Both quinic and shikimic acids have been described as precursors in the biosynthesis of several aromatic natural products in the shikimate pathway [48]. In this pathway, a reversible reduction of 3-dehydroquinic acid by quinic acid dehydrogenase occurs to produce quinic acid as a secondary metabolite [44]. Thus, a high content of quinic acid in olive fruit would lead to a lower amount of chorismic acid and L-phenylalanine and, consequently, a lesser amount of hydroxytyrosol. The biosynthesis of secoiridoids is interrelated with simple phenols, such as hydroxytyrosol, and their low availability could lead to a reduced formation of oleuropein and lucidumoside C. All these significant correlations among metabolites could be also inferred from the previously shown loading plot of PCA. Subsequent studies should, however, test this hypothesis.

## 3. Materials and Methods

### 3.1. Plant Materials

The used materials included olive fruits from 27 *cuspidata* genotypes coming from free pollination and their corresponding female parent. In addition, fruit samples from the cultivars “Arbequina”, “Frantoio”, “Picual” and “Koroneki” were also included in the experiment for comparison. The genotype acting as the female parent belongs to the wild olive Germplasm Bank preserved at the Institute of Agricultural and Fishery Research and Training, Córdoba, Spain [49]. Fruit samples (around 1 kg) were randomly collected for each plant on a common date (mid-October).

### 3.2. Chemicals and Regents

All the reagents were of analytical grade or LC-MS and used as received in the laboratory. Ethanol (EtOH) (in aqueous mixtures) was the solvent used for metabolite extraction and was supplied by Prolabo (Paris, France). Mobile phases were prepared using doubly deionized water with a conductivity of 18.2 MΩ obtained by using a Milli-Q system (Millipore, Bedford, OH, USA) (phase A) and LC-MS-grade acetonitrile (ACN) from Prolabo (Paris, France) (phase B) acidified with acetic acid (AcH), supplied by Sigma-Aldrich (St. Louis, MO, USA). Pure standards of organic acids (quinic acid), phenolic compounds (vanillin, p-coumaric acid, ferulic acid, hydroxytyrosol, tyrosol, rutin, oleuropein, luteolin, luteolin 7-O-glucoside, verbascoside, apigenin, apigenin 7-O-glucoside and pinoresinol) and pentacyclic triterpenes (maslinic, betulinic and oleanolic acids, erythrodiol and uvaol) were acquired from Sigma-Aldrich (St. Louis, MO, USA). A stock solution was prepared by dissolving an appropriate amount of each metabolite in EtOH/H_2_O (80:20 *v*/*v*) and then different dilutions were prepared to obtain diverse concentration ranges for each individual compound. All the sample extracts and standard solutions were filtered through Clarinet^TM^ 0.22 µm nylon syringe filters acquired from Bonna-Agela Technologies (Wilmington, DE, USA). Mobile phases were filtered through a Nylaflo^TM^ 0.45 µm nylon membrane filter supplied by Pall Corporation (Michigan, MI, USA). All the solutions were stored in dark flasks at −23 °C.

### 3.3. Fruit Weight and Oil Content

From each sample, three subsamples of around 25 g were randomly selected to produce dried samples sizes suitable for NMR sample holder. Fruit fresh weight was measured and, after drying in a forced-air oven at 105 °C for 42 h to ensure dehydration, oil content was determined using an NMR fat analyzer (Minispec MQone, Bruker Optik GmbH, Ettlingen, Germany) and expressed as a percentage on a dry weight basis [50].

### 3.4. Extraction and LC-MS Analysis of Fruit Metabolites

A representative sample of 50 fruits were destoned and the pulp was chopped, lyophilized and crushed to a fine powder and frozen at −23 °C. The applied metabolite-extraction procedure was the one previously reported by Olmo-García and colleagues [33,51], with a few modifications. Briefly, sample extracts were prepared by mixing 0.2 g of freeze-dried and homogenized pulp with 10 mL of EtOH/H_2_O (60:40, *v*/*v*) in a 15 mL falcon tube. After 1 min of vortex shaking, the tube was put into an ultrasound bath for 30 min and centrifuged for 5 min at 8000 rpm. Once the two phases were separated, the supernatant was transferred to a flask. The pellet was re-extracted twice by adding 10 mL of EtOH/H_2_O (80:20, *v*/*v*), applying, in both cases, the same procedure as in the first extraction. The use of EtOH/H_2_O mixtures in varying proportions ensured the effective extraction of the compounds of interest belonging to different chemical classes. All the supernatants (coming from the 3 extraction cycles) were mixed and about 1 mL of sample extract was placed in an HPLC vial after being filtered with a nylon syringe filter of 0.22 µm.

Two different LC-MS platforms were used in this study. The LC-MS system with a HRMS analyzer was used for qualitative purposes, whereas the LC platform coupled to an LR-MS was used to carry out the quantitation of the analytes of interest. For qualitative purposes, the used LC-MS platform consisted of an Acquity UPLC™ H-Class system coupled to a quadrupole-time-of-flight (QTOF) SYNAPT G2 MS (Waters, Manchester, UK). This instrument provided an accurate mass and the isotopic pattern which allowed us to predict the molecular formulae of the detected compounds, which greatly facilitates compound annotation and the identification of unknown peaks in complex matrices. Thus, the analysis of samples with HRMS helped us to describe the qualitative profiles of the samples under study. Afterwards, quantitative analyses were performed on a 1260 Infinity Agilent (Agilent Technologies, Waldbronn, Germany) coupled to an Esquire 2000 ion trap (IT) mass spectrometer (Bruker Daltonics, Bremen, Germany), which allowed us to quantify the targeted compounds by using standard calibration curves. Both MS instruments were equipped with an electrospray (ESI) interface. The selected column was an analytical Zorbax Extend C_18_ column (4.6 × 100 mm; 1.8 μm particle size) working at 40 °C. Water with 1% AcH (*v*/*v*) (phase A) and ACN with 1% AcH (*v*/*v*) (phase B) were used as mobile phases. A solvent gradient was applied for the separation of analytes and the mobile phase composition changed as follows: 0 min, 90% A and 10% B; 10 min, 75% A and 25% B; 12 min, 40% A and 60% B; 14 min, 20%A and 80%B; 18 min, 0%A and 100%B. At 20 min, the system returned to the initial conditions and the column was re-equilibrated for 3 min. The flow rate was kept constant at 1 mL/min and the injection volume was set at 10 μL. The IT MS data were acquired in full-scan mode for a mass range from 50 to 1000 *m*/*z* and the system was operated in the negative polarity mode. As far as the ESI source is concerned, the operating parameters were as follows: the nebulizer gas (nitrogen) was set at 30 psi, the dry gas flow rate was fixed at 9 L/min and dry gas temperature at 300 °C, the capillary voltage was set at +3200 V and the end-plate offset at −500 V. For HRMS analyses, these parameters were transferred to the ESI-QTOF MS system.

To operate the LC and the LR-MS systems, the Agilent ChemStation (Agilent Technologies) and Esquire Control (Bruker Daltonics) were used, respectively. The HRMS platform was controlled by means of MassLynx (Waters). The data processing was performed by using DataAnalysis v 4.0 software (Bruker Daltonics, Bremen, Germany) and Microsoft Excel v 2204.

### 3.5. Statistical Analysis

The variability for the metabolites quantified in the *cuspidata* progeny and the cultivars (“Arbequina”, “Frantoio”, “Koroneiki” and “Picual”) was studied. Correlations between fruit weight, oil content and total metabolite content as well as the cross-correlation for the metabolites quantified in the progeny were evaluated. Principal component analysis (PCA) was performed to test the relations among the different phenolic and triterpenic compounds as well as samples’ grouping by genotype. Statistix (Analytical Software, Tallahassee, FL, USA) and Unscrambler (CAMO A/S, Trondheim, Norway) were used for the statistical analysis.

## 4. Conclusions

This contribution presents the first in-depth characterization (qualitatively and quantitatively) of fruit samples from *Olea europaea* subsp. *cuspidata*. By means of a powerful LC-MS method, about 60 compounds were identified and the most representative ones were quantified. The metabolic profiles of a progeny bred through the open pollination of *cuspidata* were compared with those of a sample of cultivars, showing that the genotypes from the progeny, overall, were richer in bioactive compounds than the cultivars and, particularly, in terms of the concentrations of rutin, hydroxytyrosol glucoside, several interesting secoiridoids and the compounds of *m*/*z* 421 and 363. These results suggest that the inclusion of *cuspidata* could be very interesting for the introgression of potentially interesting compounds in breeding programs. Studies such as this one make it possible to take advantage of the potential of food metabolomics for the identification and maintenance of olive genetic diversity.

## Figures and Tables

**Table 1:** Examples of comprehensive reports describing minor components of olive fruit.

Olive Cultivar	Matrix/Matrices Considered	Analytical Platform(s) Used	Total Number of Determined Analytes	Compounds Detected in Drupes	Ref.
Frantoio and Correggilo	Olive oil, pulp and mill waste	RPLC-DAD/FLD RPLC-ESI-TQ MS	79	5 simple phenols, 5 organic acids, 12 flavonoids, 25 secoiridoids and 4 unknown compounds	[27]
Koroneiki	Olive drupes, fruit paste, unrefined oil and “final” oil	LC-PDA/ESI-LTQ- Orbitrap XL hybrid MS	52	4 simple phenols and derivatives, 25 secoiridoids and derivatives, 3 phenolic acid derivatives, 7 flavonoids, 2 triterpenes and 1 lactone	[28]
Anyvalik, Domat and Gemlik	Olive fruit and olive oil	HPLC-DAD	20	12 phenolic acids, 3 simple phenols, oleuropein, and 4 flavonoids	[29]
Arbequina, Picual, Sikitita, Arbosana, Changlot Real and Koroneiki	Olive fruit	HPLC-DAD/TOF-MS	57	18 secoiridoids, 14 flavonoids, 11 simple phenols, 9 oleosides and 5 elenolic acid glucosides	[30]
Istrska belica	Olive fruit, stones, paste, oil, pomace, and wastewater	UPLC-DAD/ESI-QTOF-HRMS	80	5 simple phenols, 4 cinnamics acids, 12 flavonoids and 24 secoiridoids	[31]
Arauco	Olive drupes and oil	GC-MS HPLC-DAD/FLD	10	3 tocopherols, squalene, 3 simple phenols and derivatives, 3 secoiridoids and 2 lignans	[32]
Picudo	Olive leaf, stem, seed, fruit skin and pulp, different types of olive oils	LC-ESI/APCI-QTOF MS GC-APCI-QTOF MS	142 in LC-MS 58 in GC-MS	12 phenolic acids and aldehydes, 4 organic acids and coumarins, 9 simple phenols and derivatives, 32 secoiridoids and derivatives, 14 flavonoids, 4 lignans, 6 pentacyclic triterpenes, 2 tocopherols and 5 sterols	[33]

APCI: Atmospheric pressure chemical ionization; DAD: Diode array detector; ESI: Electrospray ionization; FLD: Fluorescence detector; GC: Gas chromatography; HRMS: High-resolution mass spectrometry; HPLC: High-performance liquid chromatography; LC: Liquid chromatography; LTQ: Linear ion trap quadrupole; MS: Mass spectrometry; PDA: Photodiode array detector; Q: Quadrupole; RPLC: Reverse-phase liquid chromatography; TOF: Time of flight; TQ: Triple quadrupole; UPLC: Ultra-performance liquid chromatography.

**Table 2:** First qualitative exploration of progenies from *Olea europaea* subsp. *cuspidata*.

Compound	Family	Molecular Formula	Rt (min)	*m*/*z*_exp_	*m*/*z*_theo_	Error (ppm)	mSigma	*In-Source* Fragment/s	Quantified Peak Number	Standard (Quantified in Terms of)
quinic acid	organic acid	C_7_H_12_O_6_	0.90	191.0550	191.0561	5.6	7.5	-	1	quinic acid
citric acid	organic acid	C_6_H_8_O_7_	0.95	191.0190	191.0197	4.0	3.9	-		
dehydro oleuropein aglycone (A)	secoiridoids	C_19_H_20_O_8_	0.98	375.1292	375.1297	1.4	63.7	133.0133		
acyclodihydroelenolic acid hexoside (A)	secoiridoids	C_17_H_28_O_11_	1.00	407.1536	407.1559	5.6	0.27	815.3155		
oleoside/secologanoside (A)	secoiridoids	C_16_H_22_O_11_	1.00	389.1087	389.1089	0.5	17.9	345.1180		
elenolic acid glucoside (A)	secoiridoids	C_17_H_24_O_11_	1.01	403.1232	403.1246	3.4	22	223.0591		
dehydro oleuropein aglycone (B)	secoiridoids	C_16_H_24_O_10_	1.22	375.1296	375.1297	0.2	19.8	133.0139		
oleoside/secologanoside (B)	secoiridoids	C_16_H_22_O_11_	1.34	389.1083	389.1089	1.7	10.2	345.1183		
hydroxytyrosol glucoside	simple phenols	C_14_H_20_O_8_	1.34	315.1081	315.1085	1.4	11.9	153.0549	2	hydroxytyr-osol
acyclodihydroelenolic acid hexoside (B)	secoiridoids	C_17_H_28_O_11_	1.60	407.1558	407.1559	0.3	10	815.3151	3	oleuropein
dehydro acyclodihydroelenolic acid hexoside	secoiridoids	C_17_H_26_O_10_	1.62	389.1454	389.1453	−0.2	5.8	-		
oleoside/secologanoside (C)	secoiridoids	C_16_H_22_O_11_	2.36	389.1074	389.1089	4.0	6.1	345.1178	4	oleuropein
elenolic acid glucoside (B)	secoiridoids	C_17_H_24_O_11_	2.45	403.1234	403.1246	3.0	15.4	223.0601		
oxydized hydroxytyrosol	simple phenols	C_8_H_8_O_3_	3.10	151.0395	151.0401	3.5	7.1	-		
elenolic acid glucoside (C)	secoiridoids	C_17_H_24_O_11_	3.52	403.1246	403.1246	1.5	8.3	223.0598	5	oleuropein
*β*-hydroxy verbascoside	secoiridoids	C_29_H_36_O_16_	4.22	639.1929	639.1931	0.3	11.2	-	6	verbascoside
oleuropein glucoside (A)	secoiridoids	C_31_H_42_O_18_	4.61	701.2293	701.2298	0.8	8.2	-		
rutin (A)	flavonoids	C_27_H_30_O_16_	4.77	609.1463	609.1461	−0.3	14.9	-		
phenylethyl primeveroside	simple phenols	C_19_H_28_O_10_	4.96	415.1606	415.1610	0.8	6.6	-		
hydroxy decarboxymethyl oleuropein aglycone	secoiridoids	C_17_H_20_O_7_	5.14	335.1147	335.1136	−3.1	13.1	-		
demethyl oleuropein	secoiridoids	C_24_H_30_O_13_	5.73	525.1608	525.1614	1.1	5.4	1051.3298	7	oleuropein
rutin (B)	flavonoids	C_27_H_30_O_16_	5.81	609.1440	609.1461	3.4	11.2	301.0351	8	rutin
hydroxyoleuropein	secoiridoids	C_25_H_32_O_14_	6.21	555.1720	555.1719	−0.1	7.6	393.1195		
neonuzhenide	secoiridoids	C_31_H_42_O_18_	6.30	701.2292	701.2298	0.9	5.2	-	9	oleuropein
luteolin 7-O-glucoside	flavonoids	C_21_H_20_O_11_	6.48	447.0932	447.0933	0.1	6.9	285.0406	10	luteolin 7-O-glucoside
verbascoside	secoiridoids	C_29_H_36_O_15_	6.84	623.1979	623.1981	0.3	37.1	-	11	verbascoside
luteolin rutinoside	flavonoids	C_27_H_30_O_15_	7.12	593.1516	593.1512	−0.6	19.1	-		
methoxy oleuropein (A)	secoiridoids	C_26_H_34_O_14_	7.28	569.1878	569.1876	−0.4	7.5	389.1071	12	oleuropein
demethyl ligstroside	secoiridoids	C_24_H_30_O_12_	7.43	509.1666	509.1664	−0.2	10.2	347.1122	13	verbascoside
luteolin glucoside (A)	flavonoids	C_21_H_20_O_11_	7.67	447.0937	447.0933	−1.0	17.6			
dihydro oleuropein	secoiridoids	C_25_H_36_O_13_	7.71	543.2082	543.2083	0.2	21.1	525.1972 513.1981	14	oleuropein
dehydro nuzhenide	secoiridoids	C_31_H_40_O_16_	7.78	667.2244	667.2244	−0.1	11.0	310.0872	15	oleuropein
nuzhenide	secoiridoids	C_31_H_42_O_17_	7.80	685.2350	685.2349	−0.2	13.7	523.1806		
luteolin glucoside (B)	flavonoids	C_21_H_20_O_11_	7.97	447.0931	447.0933	0.4	6.1	285.0388	16	luteolin 7-O-glucoside
apigenin 7-O-glucoside	flavonoids	C_21_H_20_O_10_	8.08	431.0981	431.0984	0.7	11.7	-		
oleuropein glucoside (B)	secoiridoids	C_31_H_42_O_18_	8.12	701.2232	701.2298	−0.5	6.9	-		oleuropein
10-hydroxyoleuropein aglycon (A)	secoiridoids	C_19_H_22_O_9_	8.19	393.1189	393.1191	0.6	21.8	-		
caffeoyl 6-secologanoside	secoiridoids	C_25_H_28_O_14_	8.34	551.1385	551.1406	3.8	9.8	-	17	verbascoside
methoxy oleuropein (B)	secoiridoids	C_26_H_34_O_14_	8.85	569.1876	569.1876	−0.1	18.7	389.1069		
luteolin glucoside (C)	flavonoids	C_21_H_20_O_11_	8.92	447.0923	447.0933	2.1	7.2	-		
oleuropein	secoiridoids	C_25_H_32_O_13_	9.93	539.1768	539.1770	0.5	6.2	377.1232 307.0821	18	oleuropein
fraxamoside	secoiridoids	C_25_H_30_O_13_	10.50	537.1605	537.1614	1.7	31.3	-		
10-hydroxyoleuropein aglycon (B)	secoiridoids	C_19_H_22_O_9_	10.88	393.1180	393.1191	2.9	19.8	-		
lucidumoside C (A)	secoiridoids	C_27_H_36_O_14_	11.48	583.2031	583.2032	0.2	5.3	1167.4106 537.1594 403.1223	19	oleuropein
luteolin	flavonoids	C_15_H_10_O_6_	11.59	285.0398	285.0405	2.4	3.0	-		
elenolic acid glucoside (D)	secoiridoids	C_17_H_24_O_11_	11.70	403.1241	403.1246	1.3	11.8	223.0591		
ligstroside	secoiridoids	C_25_H_32_O_12_	11.91	523.1820	523.1821	0.2	5.5	361.1276 291.0858 259.0969	20	oleuropein
elenolic acid glucoside (E)	secoiridoids	C_17_H_24_O_11_	11.95	403.1246	403.1246	0.7	24.4	223.0594		
hydroxyoleuropein	secoiridoids	C_26_H_36_O_13_	12.03	555.2084	555.2083	−0.1	6.8	539.1779		
apigenin	flavonoides	C_15_H_10_O_5_	12.19	269.0442	269.0455	5	15.4	-		
lucidumoside C (B)	secoiridoids	C_27_H_36_O_14_	12.44	583.2026	583.2032	1.1	11.6	-		
unknown 1	-	C_19_H_24_O_7_	12.60	363.1440	363.1449	2.5	8.7	-	21	oleuropein
oleuropein aglycone (A)	secoiridoids	C_19_H_22_O_8_	12.71	377.1230	377.1242	3.3	14.5	345.0969 307.0814 275.0918	22	oleuropein
compound related to oleuropein aglycone	secoiridoids	C_20_H_26_O_8_	12.71	393.1542	393.1555	3.3	9.3	-		
compound related to oleuropein aglycone	secoiridoids	C_20_H_26_O_8_	13.10	393.1552	393.1555	0.9	6.6	-		
oleuropein aglycone (B)	secoiridoids	C_19_H_22_O_8_	13.29	377.1230	377.1242	3.2	10.7	345.0964 307.0809 275.0917	23	oleuropein
unknown 2	-	C_21_H_26_O_9_	13.58	421.1494	421.1504	2.5	9.7	-	24	oleuropein
monohydroxylated derivative of maslinic acid	pentacyclic triterpenes	C_30_H_48_O_5_	14.27	487.3420	487.3429	1.9	7.2	-		
maslinic acid	pentacyclic triterpenes	C_30_H_48_O_4_	15.78	471.3479	471.348	0.2	0.2	393.3158	25	maslinic acid
betulinic acid	pentacyclic triterpenes	C_30_H_48_O_3_	17.48	455.3529	455.3531	0.4	11.4	-	26	betulinic acid
betulinic/oleanolic acid isomer	pentacyclic triterpenes	C_30_H_48_O_3_	17.65	455.3531	455.3531	0.0	6.1	-		
oleanolic acid	pentacyclic triterpenes	C_30_H_48_O_3_	17.82	455.3528	455.3531	0.6	12.3	-	27	oleanolic acid

**Table 3:** Summary of the quantitative data obtained for the metabolites quantified in the *cuspidata* progeny and female parent, and the cultivars (“Arbequina”, “Frantoio”, “Koroneiki” and “Picual”). The compounds are ordered in the table by chemical classes and increasing concentrations in the progeny. The N column indicates the number of times each compound was quantified in each group.

Family	*cuspidata*			Cultivars		
Compound	N	Mean *	C.V. (%)	N	Mean *	C.V. (%)
Flavonoids		7195	45		1750	53
Luteolin glucoside (is B)	28	243	73	4	48	99
Luteolin 7-O-glucoside	26	500	56	4	420	73
Rutin (is B)	28	6452	50	4	1282	48
Organic acids						
Quinic acid	28	12,316	24	4	13,024	19
Pentacyclic triterpenes		13,187	31		12,612	29
Betulinic acid	28	43	76	4	15	43
Oleanolic acid	28	3588	46	4	2804	38
Maslinic acid	28	9556	27	4	9794	27
Secoiridoids		19,950	85		23,233	54
Caffeoyl 6-secologanoside	28	127	119	4	261	71
Dihydro oleuropein	28	151	56	4	755	79
Dehydro nuzhenide	28	187	126	4	72	130
*β*-hydroxy verbascoside	26	231	125	3	84	108
Neonuzhenide	28	241	68	1	322	M
Methoxy oleuropein (is A)	27	267	84	2	30	4
Oleuropein aglycone (is A)	28	418	136	4	1056	88
Oleuropein aglycone (is B)	28	562	109	4	2014	51
Demethyl ligstroside	27	994	127	1	592	M
Acyclodihydroelenolic acid hexoside (is B)	28	1023	57	4	1499	23
Oleoside/secologanoside (is C)	28	1123	66	4	314	89
Ligstroside	27	1241	161	4	1012	84
Lucidumoside C (is A)	28	1297	117	4	457	66
Demethyl oleuropein	27	1797	103	2	639	99
Elenolic acid glucoside (is C)	28	2012	57	4	1248	72
Verbascoside	18	2044	76	3	2047	85
Oleuropein	28	6237	155	4	10,831	88
Simple phenols						
Hydroxytyrosol glucoside	28	2196	73	4	1434	29
Unknowns		1009	64		212	137
Unknown 1 (*m*/*z* 363)	28	483	83	3	41	80
Unknown 2 (*m*/*z* 421)	28	526	93	4	171	89
Total		55,853	37		52,265	25

* Mean is expressed as mg·kg^−1^ of dry weight; is: isomer; M: not calculable.

**Table 4:** Cross-correlation of secondary metabolites quantified in the progeny. Significant correlations at *p* < 0.001 are highlighted.

Maslinic acid	Oleuropein	Rutin (B)	Oleanolic acid	Hydroxytyrosol glucoside	Verbascoside	Elenolic acid glucoside (C)	Demethyl oleuropein	Ligstroside	Lucidumoside C (A)	Acyclodihydroelenolic acid hexoside (B)	Oleoside/secologanoside (C)	Demethyl ligstroside	Oleuropein aglycone (B)	Oleuropein aglycone (A)	Luteolin 7-O-glucoside	Unknown 2 (*m*/*z* 421)	Unknown 1 (*m*/*z* 363)	Methoxy oleuropein (A)	Neonuzhenide	Dihydro oleuropein	Luteolin glucoside (B)	*β*-hydroxy verbascoside	Dehydro nuzhenide	Caffeoyl 6-secologanoside	Betulinic acid	
0.20	−0.61	−0.28	0.18	0.28	−0.14	0.27	0.20	−0.58	−0.69	0.28	−0.30	0.09	−0.09	−0.13	0.22	−0.23	0.31	0.19	0.07	0.04	0.04	−0.20	−0.32	0.10	−0.22	Quinic acid
	−0.48	0.09	0.69	0.00	−0.27	−0.45	−0.14	−0.34	−0.38	0.22	−0.31	−0.10	0.07	−0.01	0.53	−0.15	0.12	−0.05	−0.01	−0.15	0.17	−0.29	−0.32	0.14	0.44	Maslinic acid
		−0.27	−0.43	−0.42	0.00	−0.17	−0.39	0.65	0.62	−0.14	−0.06	−0.29	0.28	0.35	−0.48	0.05	−0.38	−0.41	−0.03	0.23	−0.33	0.05	0.12	0.12	−0.14	Oleuropein
			0.04	−0.14	−0.06	−0.17	−0.01	−0.01	0.11	−0.36	0.29	0.01	−0.43	−0.38	0.22	0.25	0.05	0.10	0.04	−0.43	0.42	0.08	0.17	−0.25	0.44	Rutin (B)
				0.06	−0.24	−0.38	−0.24	−0.21	−0.16	0.24	−0.22	−0.19	−0.09	−0.02	0.13	0.18	0.27	−0.03	−0.26	−0.39	0.08	0.07	−0.06	0.07	0.41	Oleanolic acid
					−0.17	0.28	0.37	−0.45	−0.46	0.01	0.40	0.15	−0.26	−0.25	0.15	−0.20	0.44	0.42	0.03	−0.12	0.20	−0.15	−0.20	−0.21	−0.02	Hydroxytyrosol glucoside
						0.02	−0.08	0.16	−0.04	−0.04	0.01	−0.18	0.52	0.18	−0.11	0.12	−0.42	−0.33	−0.05	0.48	−0.33	0.61	0.63	0.19	−0.32	Verbascoside
							0.57	−0.25	−0.15	−0.04	0.28	0.39	−0.41	−0.43	0.01	−0.26	0.14	0.55	0.34	0.00	0.07	−0.13	−0.12	−0.06	−0.35	Elenolic acid glucoside (C)
								−0.50	−0.47	−0.29	0.41	0.77	−0.38	−0.49	0.20	−0.40	0.15	0.85	0.06	0.03	0.16	−0.37	−0.36	−0.09	−0.14	Demethyl oleuropein
									0.64	−0.11	−0.12	−0.34	0.16	0.15	−0.56	0.22	−0.41	−0.56	−0.24	0.05	−0.38	0.33	0.38	−0.13	−0.13	Ligstroside
										−0.13	0.16	−0.37	0.07	0.35	−0.43	0.45	−0.24	−0.39	0.02	−0.24	−0.13	0.32	0.41	0.00	−0.03	Lucidumoside C (A)
											−0.42	−0.30	0.31	0.27	0.09	0.08	0.19	−0.24	−0.19	0.29	−0.10	−0.07	−0.15	0.07	−0.11	Acyclodihydroelenolic acid hexoside (B)
												0.17	−0.46	−0.35	0.00	0.14	0.40	0.39	0.11	−0.30	0.12	0.01	0.09	−0.31	0.01	Oleoside/secologanoside (C)
													−0.27	−0.39	0.17	−0.25	−0.20	0.69	−0.02	0.04	0.11	−0.27	−0.23	−0.16	−0.02	Demethyl ligstroside
														0.79	−0.11	0.01	−0.50	−0.56	−0.14	0.66	−0.30	0.14	0.18	0.30	−0.10	Oleuropein aglycone (B)
															−0.14	0.12	−0.43	−0.56	−0.24	0.23	−0.19	0.16	0.20	0.24	−0.14	Oleuropein aglycone (A)
																−0.38	0.15	0.10	0.32	−0.16	0.71	−0.41	−0.42	0.09	0.18	Luteolin 7-O-glucoside
																	0.10	−0.25	−0.22	−0.12	−0.15	0.37	0.46	−0.09	0.20	Unknown 2 (*m*/*z* 421)
																		0.34	0.19	−0.20	0.23	−0.34	−0.38	0.13	0.05	Unknown 1 (*m*/*z* 363)
																			0.24	−0.13	0.15	−0.44	−0.44	0.07	0.17	Methoxy oleuropein (A)
																				0.13	0.17	−0.30	−0.25	0.30	−0.07	Neonuzhenide
																					−0.32	−0.08	−0.01	0.19	−0.18	Dihydro oleuropein
																						−0.37	−0.29	−0.04	0.28	Luteolin glucoside (B)
																							0.98	−0.20	−0.14	*β*-hydroxy verbascoside
																								−0.25	−0.08	Dehydro nuzhenide
																									−0.14	Caffeoyl 6-secologanoside

## Data Availability

The data presented in this study are available in Table S1.

## Supplementary Materials

The following supporting information can be downloaded at: https://www.mdpi.com/article/10.3390/plants11141791/s1. Table S1: Quantitative data for each and every compound quantified in the progeny, the female parent and the cultivars.
