# Supplementary material for: Fruit Phenolic and Triterpenic Composition of Progenies of Olea europaea subsp. cuspidata, an Interesting Phytochemical Source to Be Included in Olive Breeding Programs
Source: Plants (Basel). 2022 Jul 6;11(14):1791. doi: 10.3390/plants11141791 (PMC9320664; doi:10.3390/plants11141791)
Supplement: Supplementary file 1 [file plants-11-01791-s001.zip › plants-1788053-supplementary.pdf]

Table S1. Quantitative data for each and every compound quantified in the progeny, the female parent and the cultivars (mg·kg^{-1})

Sample	Acyclodihydroelenolic acid hexoside (B)	Caffeoyl 6-secologanoside	Dehydro nuzhenide	Dihydro oleuropein	Demethyl ligstroside	Elenolic acid glucoside (C)	Ligstroside	Lucidumoside C (A)	Methoxy oleuropein (A)	Neonuzhenide	Oleoside/secologanoside (C)	Oleuropein	Demethyl oleuropein	Oleuropein aglycone (A)	Oleuropein aglycone (B)	Verbascoside	β-hydroxy verbascoside	Quinic acid	Hydroxytyrosol glucoside	Maslinic acid	Oleanolic acid	Betulinic acid	Rutin (B)	Luteolin glucoside (B)	Luteolin 7-O-glucoside	Unknown 1 (m/z 363)	Unknown 2 (m/z 421)
**315-71-G14 a**	1672.0	140.4	13.2	185.1	318.2	994.0	29.1	68.6	209.0	126.9	100.9	178.8	515.7	66.5	114.4	n.d	n.d	13136.8	1194.7	9897.9	3553.0	72.8	2855.3	10.4	n.d	821.4	106.1
**315-71-G14 b**	1544.0	129.6	12.2	170.9	293.8	918.0	26.9	63.4	193.0	117.1	93.1	165.2	476.3	61.5	105.6	n.d	n.d	12131.2	1103.3	9140.1	3281.0	67.2	2636.7	9.6	n.d	758.6	97.9
**315-77-G14 a**	2277.2	49.9	253.7	75.7	616.6	2225.2	1397.5	3825.4	208.0	153.9	1098.0	7806.8	1480.7	2249.1	1910.1	1316.4	197.6	12584.7	793.4	13772.2	5414.2	37.4	3824.4	301.5	451.3	131.0	954.5
**315-77-G14 b**	2102.8	46.1	234.3	69.9	569.4	2054.8	1290.5	3532.6	192.0	142.1	1014.0	7209.2	1367.3	2076.9	1763.9	1215.6	182.4	11621.3	732.6	12717.8	4999.8	34.6	3531.6	278.5	416.7	121.0	881.5
**315-81-G14 a**	670.7	61.3	166.4	72.8	2548.5	6233.6	486.6	1990.2	606.2	788.2	2087.9	7761.1	3718.3	13.5	31.2	1743.7	165.3	16384.1	3690.3	6379.2	1272.7	21.8	6038.1	257.9	476.2	235.0	117.5
**315-81-G14 b**	619.3	56.7	153.6	67.2	2353.5	5756.4	449.4	1837.8	559.8	727.8	1928.1	7166.9	3433.7	12.5	28.8	1610.3	152.7	15129.9	3407.7	5890.8	1175.3	20.2	5575.9	238.1	439.8	217.0	108.5
**315-85-G14 a**	1912.2	281.8	400.3	346.1	377.4	3407.4	5368.5	5209.4	97.7	165.3	2099.4	42973.9	572.9	1744.8	2293.8	4270.5	431.5	11380.6	3152.7	11043.7	3314.9	54.1	9663.9	132.1	138.3	122.4	662.4
**315-85-G14 b**	1765.8	260.2	369.7	319.7	348.6	3146.6	4957.5	4810.6	90.3	152.7	1938.6	39684.1	529.1	1611.2	2118.2	3943.5	398.5	10509.4	2911.3	10198.3	3061.1	49.9	8924.1	121.9	127.7	113.1	611.6
**315-93-G14 a**	311.9	346.3	39.9	167.4	810.0	968.1	265.1	210.0	177.8	115.4	280.7	1167.7	986.8	115.4	617.6	n.q	35.4	7721.6	447.1	9248.0	2228.3	17.7	5438.2	136.2	511.6	54.1	214.2
**315-93G14 b**	288.1	319.7	36.9	154.6	748.0	893.9	244.9	194.0	164.2	106.6	259.3	1078.3	911.2	106.6	570.4	n.q	32.6	7130.4	412.9	8540.0	2057.7	16.3	5021.8	125.8	472.4	49.9	197.8
**315-98-G14 a**	1215.5	79.0	62.9	263.1	953.5	2874.0	76.9	112.3	685.2	250.6	2501.8	590.6	4093.7	223.6	370.2	n.q	87.3	12340.3	9056.7	11711.3	4502.3	71.9	4133.2	128.9	358.7	1407.9	539.7
**315-98-G14 b**	1122.5	73.0	58.1	242.9	880.5	2654.0	71.1	103.7	632.8	231.4	2310.2	545.4	3780.3	206.4	341.8	n.q	80.7	11395.7	8363.3	10814.7	4157.7	66.3	3816.8	119.1	331.3	1300.1	498.3
**316-11-G14 a**	841.2	59.3	57.2	109.2	579.2	1374.6	37.4	79.3	210.0	127.9	422.2	319.2	2020.3	61.3	167.4	443.0	69.7	11557.4	1527.5	8155.2	2963.4	17.7	3643.5	114.4	261.0	549.0	86.3
**316-11-G14 b**	776.8	54.7	52.8	100.8	534.8	1269.4	34.6	73.3	194.0	118.1	389.8	294.8	1865.7	56.7	154.6	409.0	64.3	10672.6	1410.5	7530.8	2736.6	16.3	3364.5	105.6	241.0	507.0	79.7
**316-14-G14 a**	774.7	765.3	62.4	100.9	316.1	1925.7	382.6	2523.6	453.4	726.8	1444.3	8243.5	1647.0	243.3	220.4	n.q	67.6	9378.0	1496.3	9509.0	4267.3	43.5	7150.7	457.5	674.8	1591.9	706.0
**316-14-G14 b**	715.3	706.7	57.6	93.1	291.9	1778.3	353.4	2330.4	418.6	671.2	1333.7	7612.5	1521.0	224.7	203.6	n.q	62.4	8660.0	1381.7	8781.0	3940.7	40.1	6603.3	422.5	623.2	1470.1	652.0
**316-16-G14 a**	277.6	70.7	67.6	51.0	889.0	2664.0	38.5	87.3	345.2	367.0	685.2	485.6	2331.2	15.0	48.9	995.1	84.2	16948.7	1721.9	9935.3	3370.0	19.8	6709.8	372.2	797.5	248.5	62.4
**316-16-G14 b**	256.4	65.3	62.4	47.0	821.0	2460.0	35.5	80.7	318.8	339.0	632.8	448.4	2152.8	13.8	45.1	918.9	77.8	15651.3	1590.1	9174.7	3112.0	18.2	6196.2	343.8	736.5	229.5	57.6
**316-20-G14 a**	1387.1	241.2	25.0	46.8	125.8	2635.9	n.d	106.1	247.5	176.8	209.0	143.5	1661.6	43.7	113.0	n.d	n.d	12158.4	1132.3	7503.2	3550.9	9.4	2022.4	119.6	380.6	528.2	149.7
**316-20-G14 b**	1280.9	222.8	23.0	43.2	116.2	2434.1	n.d	97.9	228.5	163.2	193.0	132.5	1534.4	40.3	104.4	n.d	n.d	11227.6	1045.7	6928.8	3279.1	8.6	1867.6	110.4	351.4	487.8	138.3
**316-24-G14 a**	423.2	59.3	76.9	38.5	973.3	1941.3	70.7	245.4	199.6	193.4	681.1	1426.6	1523.3	43.7	115.4	532.4	139.3	16948.7	3326.3	6694.2	3311.8	21.8	2639.0	178.8	263.1	647.8	228.8
**316-24-G14 b**	390.8	54.7	71.1	35.5	898.7	1792.7	65.3	226.6	184.4	178.6	628.9	1317.4	1406.7	40.3	106.6	491.6	128.7	15651.3	3071.7	6181.8	3058.2	20.2	2437.0	165.2	242.9	598.2	211.2
**316-26-G14 a**	677.9	17.7	82.0	226.7	633.2	1146.9	343.1	610.4	188.2	522.0	545.9	2571.4	561.5	262.0	234.0	854.7	65.5	8238.3	1267.5	8309.0	1975.6	14.6	5881.1	184.0	713.3	296.3	166.4
**316-26-G14 b**	626.1	16.3	75.8	209.3	584.8	1059.1	316.9	563.6	173.8	482.0	504.1	2374.6	518.5	242.0	216.0	789.3	60.5	7607.7	1170.5	7673.0	1824.4	13.4	5430.9	170.0	658.7	273.7	153.6
**316-28-G14 a**	800.6	340.0	605.2	229.8	241.2	1151.1	569.8	1250.8	126.9	214.2	672.8	5774.0	443.0	1574.3	2053.6	4323.5	773.2	19207.2	4125.9	12111.6	6577.8	41.5	3156.8	263.1	477.3	190.1	988.8
**316-28-G14 b**	739.4	314.0	558.8	212.2	222.8	1062.9	526.2	1155.0	117.1	197.8	621.2	5332.0	409.0	1453.7	1896.4	3992.5	714.0	17736.8	3810.1	11184.4	6074.2	38.3	2915.2	242.9	440.7	175.5	913.2
**316-30-G14 a**	664.4	8.3	479.3	121.7	595.8	1515.0	6297.0	2240.8	44.7	212.5	1385.0	7884.8	123.7	269.3	967.0	4489.9	499.1	13940.6	2124.3	13565.2	5757.4	57.2	12385.1	47.8	142.6	83.2	394.1
**316-30-G14 b**	613.6	7.7	442.7	112.3	550.2	1399.0	5815.0	2069.2	41.3	196.3	1279.0	7281.2	114.3	248.7	893.0	4146.1	460.9	12873.4	1961.7	12526.8	5316.6	52.8	11436.9	44.2	131.6	76.8	363.9
**316-32-G14 a**	893.2	68.6	227.7	332.7	2294.8	2699.3	596.8	1227.0	741.4	264.1	2089.0	6150.4	5978.9	227.7	230.8	2003.7	322.3	15780.0	2479.9	5829.1	1502.5	34.3	14375.2	133.1	234.0	401.4	628.0
**316-32-G14 b**	824.8	63.4	210.3	307.3	2119.2	2492.7	551.2	1133.0	684.6	243.9	1929.0	5679.6	5521.2	210.3	213.2	1850.3	297.7	14572.0	2290.1	5382.9	1387.5	31.7	13274.8	122.9	216.0	370.6	580.0
**316-33-G14 a**	891.1	97.7	55.1	281.8	6868.9	3214.0	350.4	175.4	1013.8	100.9	1619.0	1962.1	8392.2	70.7	182.0	722.7	60.3	14484.4	3310.7	14317.0	5815.6	61.3	5752.2	376.4	838.1	180.9	158.0
**316-33-G14 b**	822.9	90.3	50.9	260.2	6343.1	2968.0	323.6	162.0	936.2	93.1	1495.0	1811.9	7749.8	65.3	168.0	667.3	55.7	13375.6	3057.3	13221.0	5370.4	56.7	5311.8	347.6	773.9	167.1	146.0
**316-35-G14 a**	1997.5	120.6	777.8	183.0	988.8	3949.2	3667.4	3822.3	209.0	326.5	2620.3	23539.0	1103.2	314.0	422.2	3509.3	932.7	12047.1	2108.7	12178.1	6261.7	78.0	9954.0	56.1	n.q	335.9	1545.1
**316-35-G14 b**	1844.5	111.4	718.2	169.0	913.2	3646.8	3386.6	3529.7	193.0	301.5	2419.7	21737.0	1018.8	290.0	389.8	3240.7	861.3	11124.9	1947.3	11245.9	5782.3	72.0	9192.0	51.9	n.q	310.1	1426.9
**316-37-G14 a**	619.7	42.6	184.0	291.1	1546.2	2549.6	94.6	164.3	217.7	167.4	760.1	1070.0	3214.0	128.9	431.5	3592.5	242.3	10482.2	1680.3	5331.1	1087.6	8.5	3931.5	175.7	735.1	235.0	281.8

Table S1. Quantitative data for each and every compound quantified in the progeny, the female parent and the cultivars (mg·kg^{-1})

Sample	Acyclodihydroelenolic acid hexoside (B)	Caffeoyl 6-secologanoside	Dehydro nuzhenide	Dihydro oleuropein	Demethyl ligstroside	Elenolic acid glucoside (C)	Ligstroside	Lucidumoside C (A)	Methoxy oleuropein	Neonuzhenide	Oleoside/secologanoside (C)	Oleuropein	Demethyl oleuropein	Oleuropein aglycone (A)	Oleuropein aglycone (B)	Verbascoside	β-hydroxy verbascoside	Quinic acid	Hydroxytyrosol glucoside	Maslinic acid	Oleanolic acid	Betulinic acid	Rutin (B)	Luteolin glucoside (B)	Luteolin 7-O-glucoside	Unknown 1 (m/z 363)	Unknown 2 (m/z 421)
**316-37-G14 b**	572.3	39.4	170.0	268.9	1427.8	2354.4	87.4	151.7	201.1	154.6	701.9	988.0	2968.0	119.1	398.5	3317.5	223.7	9679.8	1551.7	4922.9	1004.4	7.9	3630.5	162.3	678.9	217.0	260.2
**316-38-G14 a**	2352.0	30.2	167.4	142.5	462.7	2121.2	183.0	915.0	141.4	120.6	590.6	1767.7	365.0	641.6	555.3	810.0	153.9	12549.3	3570.7	9913.5	3949.2	68.6	9370.7	791.3	1034.6	607.2	484.5
**316-38-G14 b**	2172.0	27.8	154.6	131.5	427.3	1958.8	169.0	845.0	130.6	111.4	545.4	1632.3	337.0	592.4	512.7	748.0	142.1	11588.7	3297.3	9154.5	3646.8	63.4	8653.3	730.7	955.4	560.8	447.5
**316-43-G14 a**	1483.8	23.9	96.7	100.5	1211.4	1297.7	236.0	671.7	298.4	130.2	1007.6	1055.4	629.1	71.7	339.0	n.q	98.8	14592.6	1580.5	10180.7	6180.6	101.9	9291.7	183.0	218.6	976.4	1681.4
**316-43-G14 b**	1370.2	22.1	89.3	92.9	1118.6	1198.3	218.0	620.3	275.6	120.2	930.4	974.6	580.9	66.3	313.0	n.q	91.2	13475.4	1459.5	9401.3	5707.4	94.1	8580.3	169.0	201.8	901.6	1552.6
**316-4-G14 a**	1124.0	19.8	997.2	94.6	237.1	1171.9	2619.3	2796.0	42.6	130.0	1838.4	5150.1	99.8	1261.3	1588.4	5065.9	1197.8	14070.6	1159.4	14302.4	5970.5	40.6	13887.6	74.2	118.5	316.1	1486.9
**316-4-G14 b**	1038.0	18.2	920.8	87.4	218.9	1082.1	2418.7	2582.0	39.4	120.0	1697.6	4755.9	92.2	1164.7	1466.8	4678.1	1106.2	12993.4	1070.6	13207.6	5513.5	37.4	12824.4	68.6	109.5	291.9	1373.1
**316-5-G14 a**	785.0	164.3	114.5	208.0	1724.0	2222.1	934.8	244.4	252.7	222.5	411.8	3819.2	1882.0	146.6	196.5	1227.0	111.3	14338.8	2556.9	6699.4	1646.0	22.0	5211.5	471.0	596.8	251.6	226.7
**316-5-G14 b**	725.0	151.7	105.7	192.0	1592.0	2051.9	863.2	225.6	233.3	205.5	380.2	3526.8	1738.0	135.4	181.5	1133.0	102.7	13241.2	2361.1	6186.6	1520.0	20.4	4812.5	435.0	551.2	232.4	209.3
**316-6-G14 a**	1282.1	54.1	29.1	88.4	736.2	1706.3	162.2	144.5	295.3	153.9	1702.2	2568.3	2565.2	144.5	183.0	n.q	32.2	13385.3	2780.4	8735.4	2941.6	27.0	7096.6	520.9	1016.9	1207.2	232.9
**316-6-G14 b**	1183.9	49.9	26.9	81.6	679.8	1575.7	149.8	133.5	272.7	142.1	1571.8	2371.7	2368.8	133.5	169.0	n.q	29.8	12360.7	2567.6	8066.6	2716.4	25.0	6553.4	481.1	939.1	1114.8	215.1
**316-87-G14 a**	1344.5	136.2	196.5	184.0	480.4	1349.7	2358.3	2056.7	101.9	205.3	987.8	7722.6	163.2	608.3	453.4	471.0	162.2	13384.3	1112.6	7924.3	2107.7	22.9	4799.7	41.6	104.0	639.5	1646.0
**316-87-G14 b**	1241.5	125.8	181.5	170.0	443.6	1246.3	2177.7	1899.3	94.1	189.5	912.2	7131.4	150.8	561.7	418.6	435.0	149.8	12359.7	1027.4	7317.7	1946.3	21.1	4432.3	38.4	96.0	590.5	1520.0
**316-89-G14 a**	432.6	82.1	37.7	71.7	907.7	2055.7	71.7	807.9	228.8	304.7	2664.0	3621.6	2701.4	72.8	211.1	n.q	36.4	13049.5	2572.5	11150.8	3291.0	40.6	6181.6	403.4	1050.2	1063.5	230.8
**316-89-G14 b**	399.4	75.9	34.9	66.3	838.3	1898.3	66.3	746.1	211.2	281.3	2460.0	3344.4	2494.6	67.2	194.9	n.q	33.6	12050.5	2375.5	10297.2	3039.0	37.4	5708.4	372.6	969.8	982.1	213.2
**316-8-G14 a**	848.5	73.8	14.6	151.6	359.8	872.4	84.2	151.8	131.0	213.2	737.2	545.9	1578.4	294.3	506.4	n.q	6.2	10953.3	2681.6	9193.9	2541.3	26.0	4781.0	294.3	675.9	639.5	163.2
**316-8-G14 b**	783.5	68.2	13.4	140.0	332.2	805.6	77.8	140.2	121.0	196.8	680.8	504.1	1457.6	271.7	467.6	n.q	5.8	10114.7	2476.4	8490.1	2346.7	24.0	4415.0	271.7	624.1	590.5	150.8
**316-91-G14 a**	1729.2	213.2	75.9	141.4	115.9	2159.7	7662.3	5360.2	29.0	335.9	978.5	31830.4	85.3	1055.4	1223.8	1880.0	241.2	13067.2	693.5	15041.7	6189.9	41.6	10599.7	531.3	685.2	298.4	793.4
**316-91-G14 b**	1596.8	196.8	70.1	130.6	107.1	1994.3	7075.7	4949.8	26.8	310.1	903.5	29393.6	78.7	974.6	1130.2	1736.0	222.8	12066.8	640.5	13890.3	5716.1	38.4	9788.3	490.7	632.8	275.6	732.6
**Female parent a**	106.1	38.3	74.9	86.3	n.d	149.3	447.1	657.2	n.d	190.3	470.0	3442.8	n.d	209.0	586.4	n.q	35.4	4461.8	585.4	9584.9	2920.8	167.4	7060.2	321.3	567.7	7.7	363.9
**Female parent b**	97.9	35.3	69.1	79.7	n.d	137.9	412.9	606.8	n.d	175.7	434.0	3179.2	n.d	193.0	541.6	n.q	32.6	4120.2	540.6	8851.1	2697.2	154.6	6519.8	296.7	524.3	7.1	336.1
**Arbequina a**	1610.7	142.5	14.3	65.5	n.d	93.6	18.7	97.7	30.4	n.d	94.6	245.4	199.3	676.9	1313.3	742.9	n.d	10776.5	1889.3	11799.7	3741.2	18.7	2064.0	118.5	829.8	36.4	92.5
**Arbequina b**	1487.3	131.5	13.3	60.5	n.d	86.4	17.3	90.3	28.0	n.d	87.4	226.6	184.1	625.1	1212.7	686.1	n.d	9951.5	1744.7	10896.3	3454.8	17.3	1906.0	109.5	766.2	33.6	85.5
**Frantoio a**	2046.3	235.0	215.2	1548.3	615.6	2076.5	1367.3	351.5	32.2	334.8	254.8	6979.1	1129.2	795.4	3065.3	4177.9	188.2	12606.5	1109.5	6806.5	1382.9	5.4	603.1	16.6	266.2	80.1	412.8
**Frantoio b**	1889.7	217.0	198.8	1429.7	568.4	1917.5	1262.7	324.5	29.8	309.2	235.2	6444.9	1042.8	734.6	2830.7	3858.1	173.8	11641.5	1024.5	6285.5	1277.1	5.0	556.9	15.4	245.8	73.9	381.2
**Koroneiki a**	1309.1	152.9	60.3	927.5	n.d	1980.8	2086.9	642.6	n.d	n.d	751.8	23432.9	n.d	395.1	1044.0	1464.0	70.7	14005.1	1840.4	12990.2	3669.5	19.0	1068.9	10.4	106.1	n.d	118.5
**Koroneiki b**	1208.9	141.1	55.7	856.5	n.d	1829.2	1927.1	593.4	n.d	n.d	694.2	21639.1	n.d	364.9	964.0	1352.0	65.3	12932.9	1699.6	11995.8	3388.5	17.6	987.1	9.6	97.9	n.d	109.5
**Picual a**	1268.6	556.3	7.9	598.9	n.d	1040.8	735.1	810.0	n.d	n.d	204.8	14391.9	n.d	2524.6	2954.1	n.q	1.9	16782.4	1124.0	9137.8	2866.7	17.7	1594.0	54.6	545.9	12.5	85.3
**Picual b**	1171.4	513.7	7.3	553.1	n.d	961.2	678.9	748.0	n.d	n.d	189.2	13290.1	n.d	2331.4	2727.9	n.q	1.7	15497.6	1038.0	8438.2	2647.3	16.3	1472.0	50.4	504.1	11.5	78.7

SD values have not been included to contain the size of the table. Coefficient of variation (%) were below 4.3% in every case.
